# 661W is a retinal ganglion precursor-like cell line in which glaucoma-associated optineurin mutants induce cell death selectively

**DOI:** 10.1038/s41598-017-17241-0

**Published:** 2017-12-04

**Authors:** Zuberwasim Sayyad, Kapil Sirohi, Vegesna Radha, Ghanshyam Swarup

**Affiliations:** 1 CSIR-Centre for Cellular and Molecular Biology, Hyderabad, 500007 India; 2 Present Address: Department of medicine, National Jewish Health, Denver, 80206 Colorado, USA

## Abstract

A photoreceptor cell line, 661W, derived from a mouse retinal tumor that expresses several markers of cone photoreceptor cells has been described earlier. However, these cells can be differentiated into neuronal cells. Here, we report that this cell line expressed certain markers specific to retinal ganglion cells such as Rbpms, Brn3b (Pou4f2), Brn3c (Pou4f3), Thy1 and γ-synuclein (Sncg), and some other markers of neuronal cells (beta-III tubulin, NeuN and MAP2). These cells also expressed Opn1mw, a cone-specific marker and nestin, a marker for neural precursor cells. Two glaucoma-associated mutants of OPTN, E50K and M98K, but not an amyotrophic lateral sclerosis-associated mutant, E478G, induced cell death selectively in 661W cells. However, in a motor neuron cell line, NSC34, E478G mutant of OPTN but not E50K and M98K induced cell death. We conclude that 661W is a retinal ganglion precursor-like cell line, which shows properties of both retinal ganglion and photoreceptor cells. We suggest that these cells could be utilized for exploring the mechanisms of cell death induction and cytoprotection relevant for glaucoma pathogenesis. RGC-5 cell line which probably arose from 661W cells showed expression of essentially the same markers of retinal ganglion cells and neuronal cells as seen in 661W cells.

## Introduction

There are several types of cells in the retina organized in multiple layers which are affected by various disorders of retina that contribute to blindness worldwide. Defects in photoreceptor cells are involved in the pathogenesis of retinal dystrophy and retinitis pigmentosa, whereas retinal ganglion cells (RGCs) are affected in optic neuropathy and glaucoma^1–3^. Glaucoma is a leading cause of irreversible blindness, characterized by increased optic cup to disc ratio, tunnel vision and degeneration of RGCs and their axons^4^. In addition to degeneration of RGCs, glaucoma in adults is associated with loss of cone photoreceptor cells in humans and experimental animal models^5–8^. Genetic as well as environmental factors contribute to development of various types of glaucoma^9,10^. Increased intraocular pressure (IOP) is a major risk factor for glaucoma in adults. Mutations in several genes including *OPTN*, which codes for the protein optineurin, are associated with glaucoma^11–19^. Certain mutations of OPTN are associated with NTG (normal tension glaucoma), a sub-group of adult onset primary open angle glaucoma in which IOP remains in the normal range^20^. Another set of mutations of OPTN are associated with amyotrophic lateral sclerosis (ALS), a motor neuron disease, suggesting, therefore, that deleterious effects of OPTN mutations are cell/tissue type dependent^21^.

Cell lines are often used to facilitate investigations to understand the molecular basis of eye diseases so that preventive or therapeutic interventions can be designed. A mouse photoreceptor cell line, 661W, has been described which is derived from a retinal tumor formed in a transgenic mouse expressing SV40 large T-antigen under the control of IRBP (interphotoreceptor retinoid-binding protein) promoter^22^. This cell line expresses several markers of cone photoreceptor cells but not of rod cells. Sensitivity of this cell line to light has also been reported^23^. However, morphologically, these cells show processes generally seen in neuronal cells and do not form outer segment-like membranes seen in photoreceptor cells^22^. Treatment of these cells with staurosporine (STSN) induces differentiation into neuronal cells that show increased number and branching of neurites per cell^24^. Although expression of several molecular markers for cone and rod photoreceptors and retinal pigment epithelium (RPE), has been tested, the markers for RGCs have not been examined in this cell line.

A retinal ganglion cell line, RGC-5, of rat origin was described in 2001, which was shown to express several markers specific for RGCs^25^. This cell line has been used extensively by several research groups. Re-characterization of this cell line revealed that this cell line is of mouse origin and does not express some of the markers of RGCs that were tested (Brn3, Thy1, neurofilaments) but it expresses several markers of neuronal cells (Map1b, MAP2, PGP9.5 and beta-III tubulin)^26,27^. Expression of Thy1 in this cell line is controversial^25,27^. cDNAs coding for mouse *Optn*, *Tbc1d17* and *Tbk1* have been cloned by RT-PCR using RNA from this cell line, further supporting that it is of mouse origin^28,29^. In addition, RGC-5 cells express nestin, a marker for neural precursor cells, indicating, therefore that it is a neuronal precursor cell line^27^. These cells also show expression of a cone-specific opsin, OPN1SW, which is expressed in 661W cells^27^. On the basis of these and some other observations, it was suggested that RGC-5 cells probably originated from 661W photoreceptor cells that were also being used in the laboratory of the investigator who originally described RGC-5 cell line^30^. However, RGC-5 cells show some interesting properties. A glaucoma-associated mutant of OPTN, E50K, induces significantly more cell death than wild type OPTN when expressed in RGC-5 cells but not in many other cell lines tested such as HeLa, COS-7, Neuro2a, IMR32, and SH-SY5Y^31–34^. The E50K mutant of OPTN is causatively associated with NTG in humans^12,35^ and it has been shown to induce degeneration of RGCs in transgenic mouse models^36–38^. Another glaucoma-associated variant of OPTN, M98K, induces cell death and Tbk1-dependent phosphorylation selectively in RGC-5 cells but not in IMR32 or HeLa cells^29,39^. These RGC-like properties of RGC-5 cells cannot be explained by the molecular marker analysis that has been done during re-characterization. The expression of Brn3 family of transcription factors (Brn3a, Brn3b and Brn3c), which are crucial for the differentiation of RGCs from multipotent retinal precursor cells (RPCs), has not been analyzed adequately and relied entirely upon an antibody (used for immunostaining of cells) that was believed to recognize all three Brn3 proteins in human cells. Specificity of this Brn3 antibody was not demonstrated by western blots^27^. Therefore, a more extensive investigation of RGC-5 cells using additional molecular markers is needed to resolve these issues^40–46^.

Here, we have re-characterized the 661W cell line by using various molecular markers of RGCs and neuronal cells. Our results show that these cells express certain markers of RGCs (Rbpms, Brn3b, Brn3c, Thy1 and γ-synuclein) and of neuronal cells. These cells also express nestin a neural precursor cell marker. In addition, we examined the effect of expression of disease-associated mutants of OPTN in these cells. Two glaucoma-associated mutants of OPTN, E50K and M98K, induced significantly more cell death than wild type (WT) OPTN selectively in 661W cells but an ALS-associated mutant of OPTN, E478G, did not induce cell death. However, in a cell culture model of ALS, NSC34 cell line, the E478G mutant of OPTN induced cell death but E50K and M98K mutants did not. We conclude that 661W is a RGC precursor-like cell line with properties of both retinal ganglion and photoreceptor cells. In addition, we find that RGC-5 cells show very similar pattern of expression of RGC-specific and other markers that are seen in 661W cells.

## Results

### 661W cells express RGC specific markers

**Figure 1:**
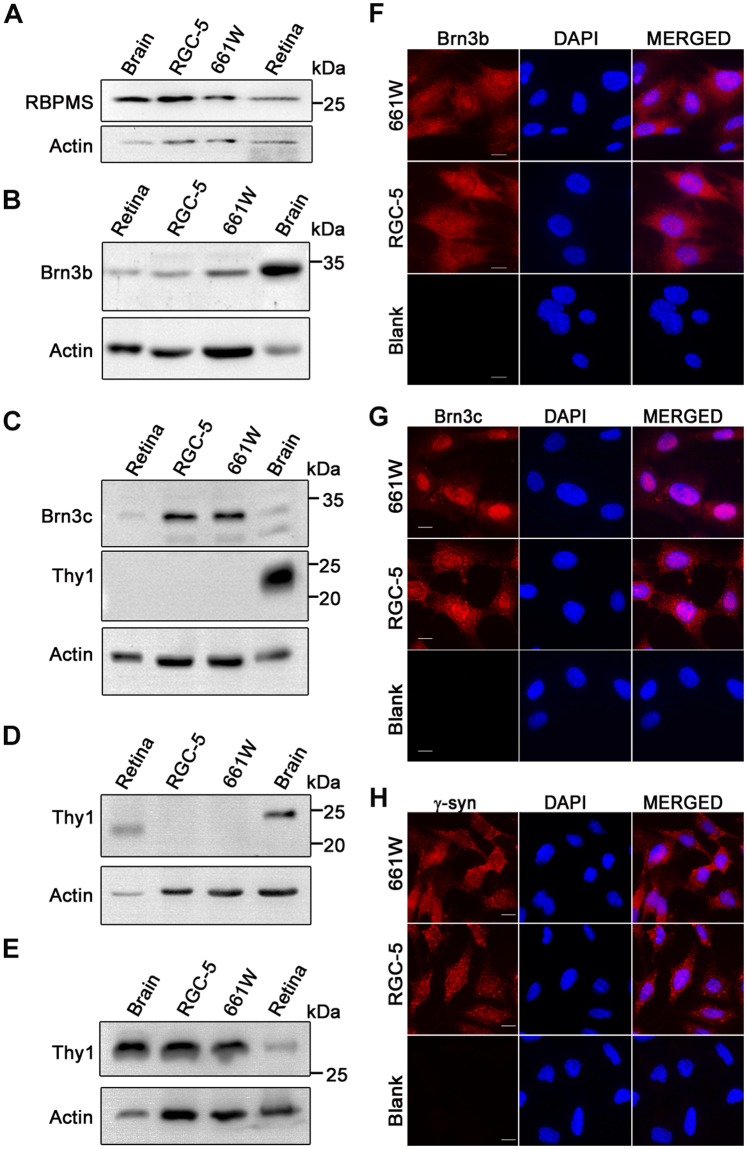
Expression of RGC-specific markers in 661W cells. 661W and RGC-5 cell lysates were subjected to western blotting for the expression of RBPMS (**A**), Brn3b (**B**), Brn3c (**C**) and Thy-1 (**C**,**D** and **E**). For Thy1 three different antibodies sourced from CST (**C**), R & D systems (**D**) and Santa Cruz (**E**) were used. Mouse retina and brain were used as positive controls. Immunofluorescence staining of 661W and RGC-5 cells for Brn3b (**F**), Brn3c (**G**) and γ Synuclein (**H**) is shown. Staining of nuclear DNA with DAPI is shown in blue. Blank indicates negative control where cells were processed similarly without addition of primary antibody. Panels show images captured using a fluorescence microscope at 40X magnification. Scale bar, 10 µm.

The pattern of expression of genes and proteins determines the nature and characteristics of cells. Retrograde labeling of RGCs has been a gold standard for identification of RGCs in retina^47^. Further, immunostaining studies of mouse retina have revealed the expression of certain proteins in the ganglion cells, which show a strong co-relation with retrograde labeling, indicating that these proteins can be used as markers for identification of RGCs^40,45,48^. Hence, within the retina RGCs can be characterized based on the expression of molecular markers like Rbpms, Brn3 family members, γ-Synuclein and Thy1. To determine the expression of these RGC specific proteins, immunoblotting and/or immunofluorescence staining were performed. Expression of Rbpms was observed as a band of about 25 kDa in 661W as well as RGC-5 cells by immunoblotting (Fig. 1A). A similar band was seen in mouse retina, which was used as a positive control. Brn3b and Brn3c proteins were detected in 661W cells by western blotting using specific antibodies. Expression of Brn3b and Brn3c was also seen in these cell lines and a corresponding protein band was seen in retina as well as brain (Fig. 1B and C). Expression of Brn3c in 661W and RGC-5 cells was much higher than in retina or brain (Fig. 1C). Immunofluorescence staining of 661W and RGC-5 cells grown on coverslips showed that Brn3b and Brn3c were expressed in all the cells (Fig. 1F and G). Brn3c showed prominent nuclear localization with some cytoplasmic staining, whereas Brn3b showed predominantly cytoplasmic staining with some nuclear localization. Expression of Brn3a was not seen (data not shown). Expression of γ-synuclein protein was observed by immunostaining of 661W as well as RGC-5 cells and it was observed that its localisation was similar to that described earlier (Fig. 1H)^42^. Elimination of primary antibody abolished the signal as indicated in blank panels (Fig. 1F,G and H).

Expression of Thy1 protein was examined by using 3 different antibodies. With two different antibodies Thy1 protein could not be detected in 661W or RGC-5 cells by western blotting although it could be seen in brain (Fig. 1C and D). One of these two antibodies could not detect Thy1 even in retina but it could detect in brain (Fig. 1C,D). However, another antibody showed expression of Thy1 in 661W as well as RGC-5 cells and the band position was the same as in retina and brain (Fig. 1E). These results with various antibodies indicate that Thy1 is expressed in different forms possibly generated by differential glycosylation or alternate splicing^49^.

### Expression of neuronal markers in 661W cells

**Figure 2:**
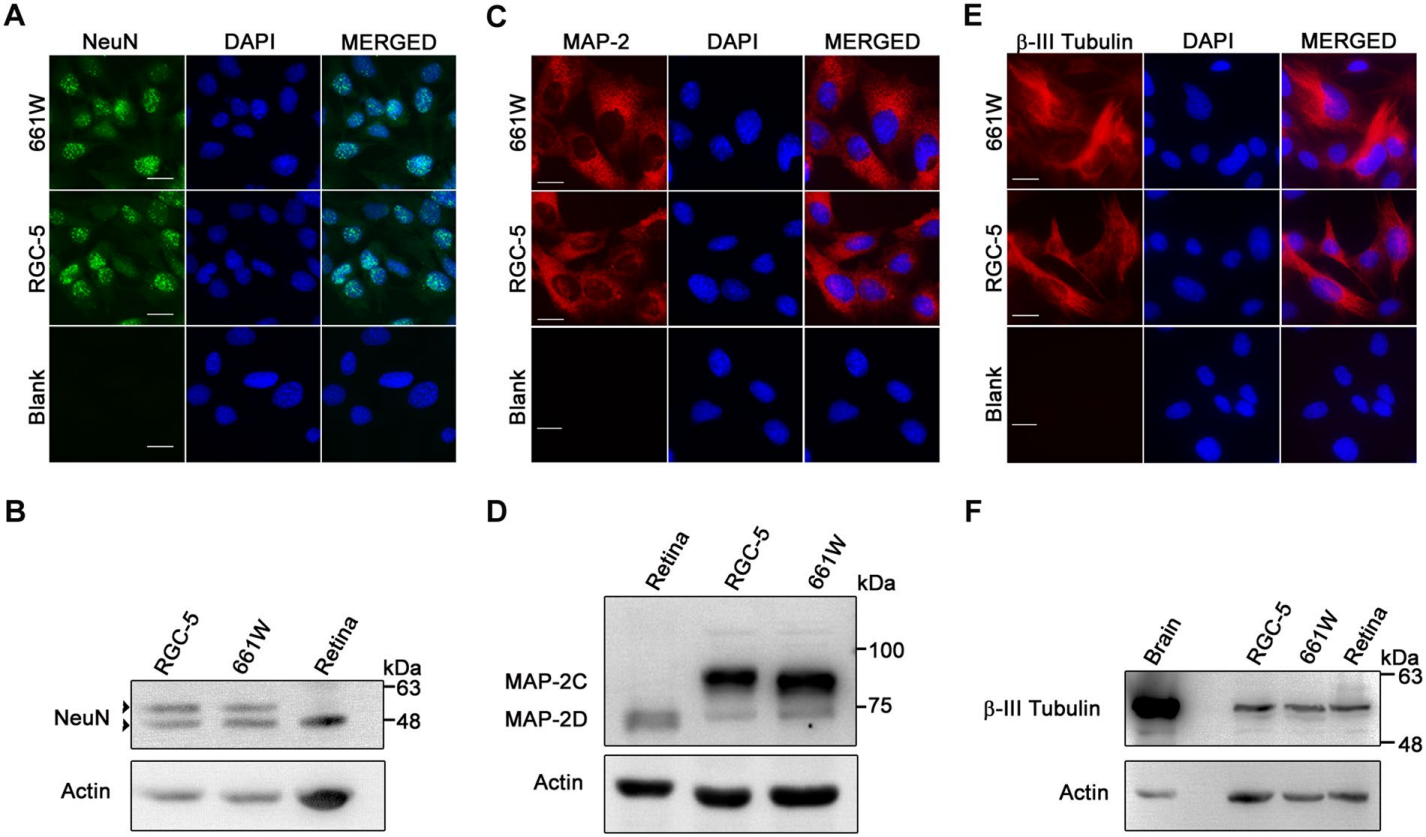
Expression of neuronal markers in 661W cells. (**A**,**C**,**E**) Immunostaining of 661W and RGC-5 cells for NeuN (**A**), MAP2 (**C**) and beta-III tubulin (**E**). Blank indicates negative control where cells are processed similarly without addition of primary antibody. Scale bar, 10 µm. (**B**,**D**,**F**) 661W and RGC-5 cell lysates were resolved on SDS PAGE and blotted for the expression of NeuN (**B**), MAP-2 (**D**) and beta-III tubulin (**F**). Arrow heads indicate alternate isoforms of NeuN. Mouse retina was used as positive control.

Neuronal nuclei (NeuN, also known as Rbfox3) is a widely used marker of neuronal cells which is generally detected by immunostaining of cells with a monoclonal antibody^50,51^. In the retina NeuN is expressed only in RGCs and not in other cells^36,52^. NeuN expression was seen in the nuclei of 661W as well as RGC-5 cells (Fig. 2A) and also by western blotting (Fig. 2B). Another neuronal marker MAP2 was expressed in 661W as well as RGC-5 cells by immunostaining of cells as well as western blotting (Fig. 2C and D). Expression of MAP-2 isoforms, MAP-2C and MAP-2D was observed in both the cell lines (Fig. 2D). Expression of beta-III tubulin, a neuronal marker, which has also been used as RGC-specific marker, was examined by western blot and also by immunostaining of cells. Both 661W and RGC-5 cells showed expression of beta-III tubulin (Fig. 2E,F).

### Expression of markers of cone photoreceptors and neuronal precursor cells in 661W and RGC-5 cells

**Figure 3:**
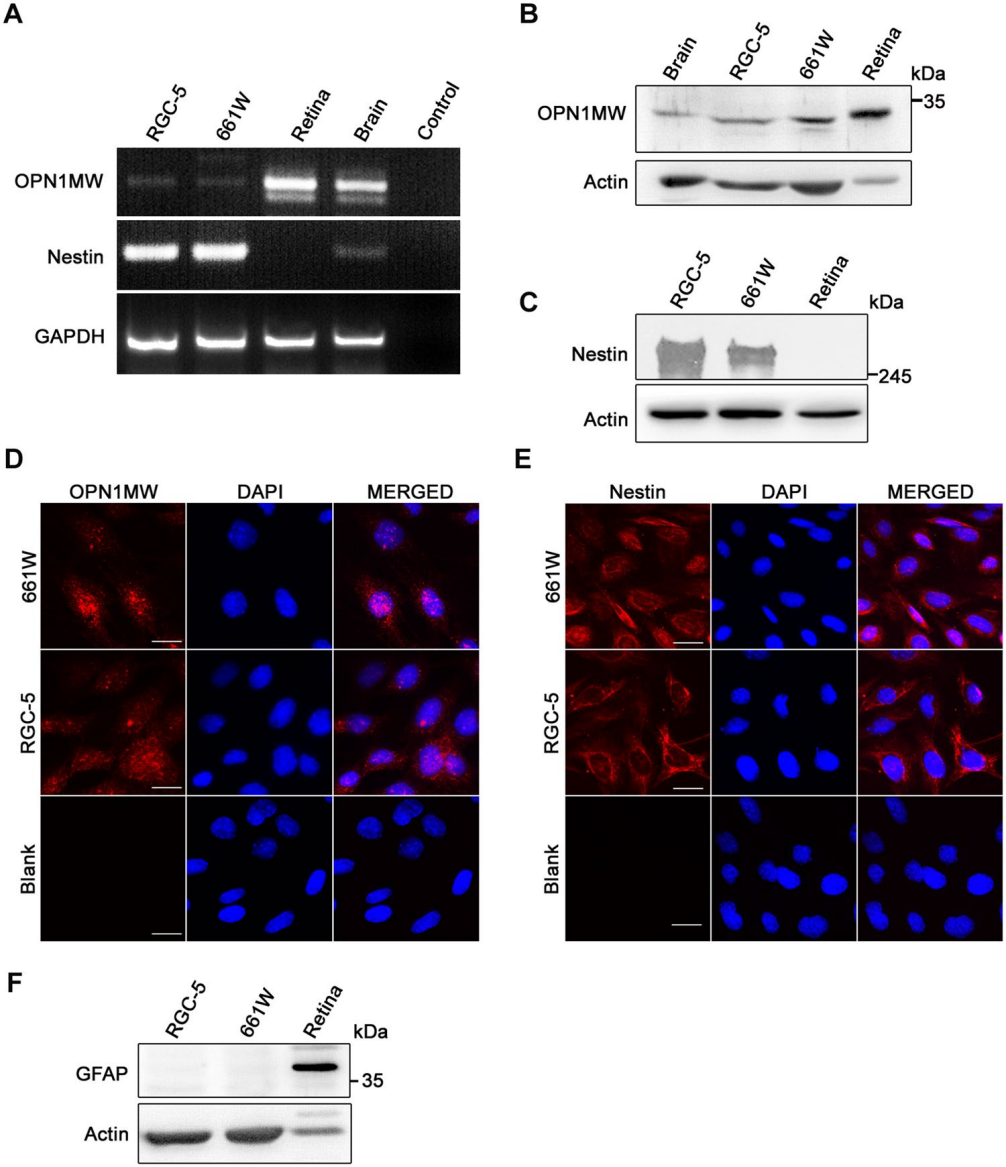
Expression of a cone-specific marker and nestin in 661W cells. (**A**) Semi quantitative PCR analysis for mRNA expression of cone-specific marker OPN1MW and neuronal precursor marker nestin. (**B**,**C**) Western blot analysis of 661W and RGC-5 cells showing the expression of OPN1MW and nestin, respectively. (**D**,**E**) Immunostaining of 661W and RGC-5 cells for OPN1MW and nestin respectively. Scale bar, 10 µm. (**F**) Expression analysis of GFAP, a glial cell specific marker by western blotting.

To ascertain the photoreceptor nature of 661W cells used in this study we examined the expression of cone specific marker Opn1mw by RTPCR, western blot and immunofluorescence staining^22^. Expression of Opn1mw mRNA (Fig. 3A) as well as protein (Fig. 3B) was seen in these cells. The level of Opn1mw mRNA and protein in these cells was much lower compared to that seen in mouse retina. Immunofluorescence staining showed the presence of Opn1mw both in the nucleus and cytoplasm (Fig. 3D). RGC-5 cells also showed expression of Opn1mw (Fig. 3A,B and D). Expression of neuronal precursor marker nestin was seen in 661W as well as RGC-5 cells by RTPCR (Fig. 3A) and also by western blot (Fig. 3C). Immunostaining of these cells revealed expression of nestin as a network of filaments in all the cells (Fig. 3E). Western blotting was performed to analyse expression of GFAP (Glial Fibrillary Acidic Protein), a marker of astrocytes in retina^53^. Neither 661W nor RGC-5 cells expressed GFAP when examined by western blot, whereas positive control (retina) showed its expression, indicating that these cells do not show features of glial cells (Fig. 3F).

### Morphological analysis and differentiation of 661W cells

**Figure 4:**
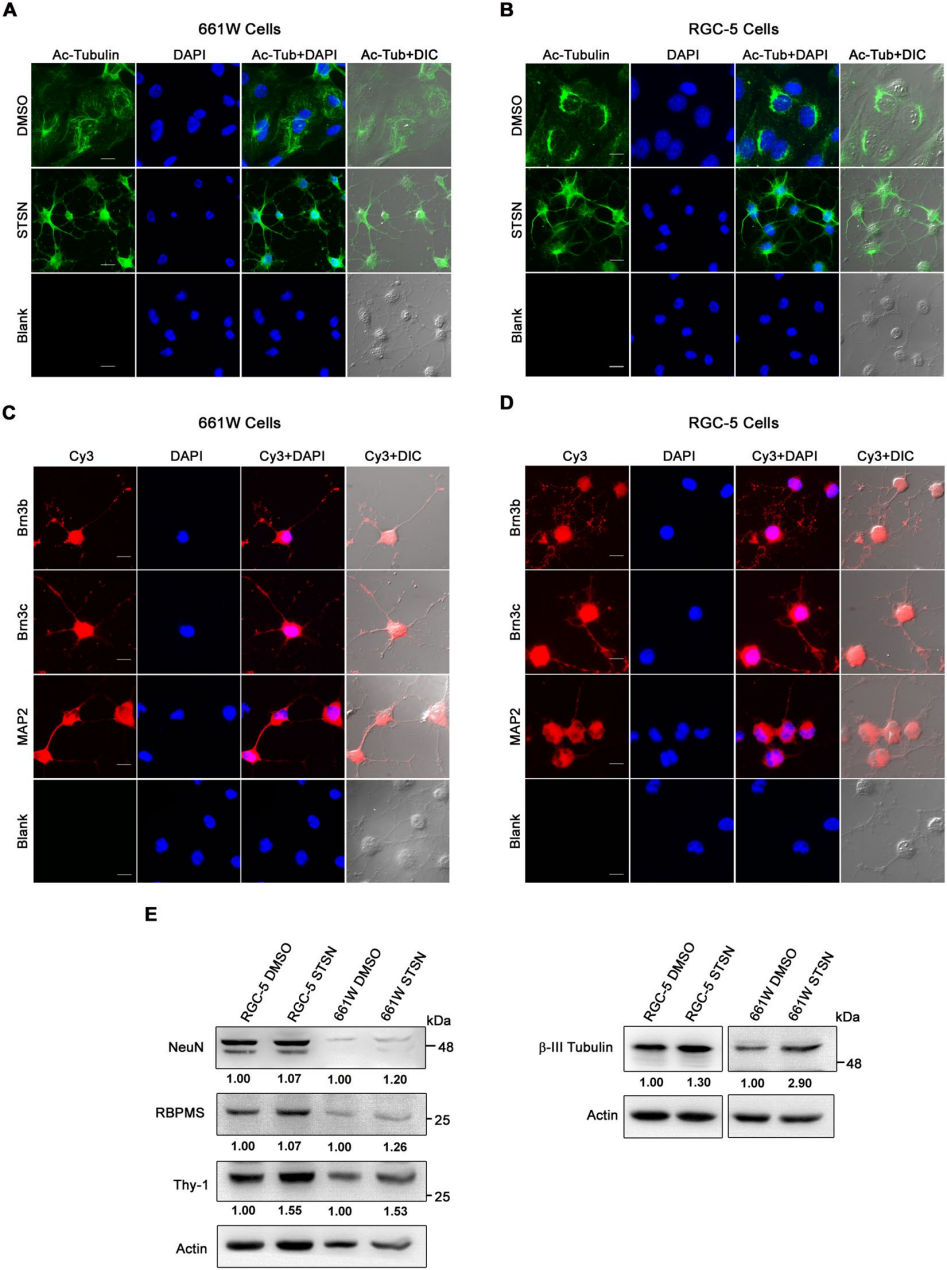
Morphological analysis and differentiation of 661W and RGC-5 cells. 661W (**A**,**C**) and RGC-5 cells (**B**,**D**) were treated with 316 nM staurosporine (STSN) or vehicle (DMSO) for 24 hours, fixed and stained with specific antibody for acetylated tubulin (**A**,**B**). Staurosporine treatment induced neurite formation in 661W as well as RGC-5 cells. Scale bar: 10 µm. (**C**,**D**) Immunostaining of 661W and RGC-5 cells for RGC markers Brn3b, Brn3c and neuronal marker MAP-2 with antibodies. Scale bar, 10 µm. (**E**) Western blots showing the effect of differentiation with staurosporine on the level of Thy1, RBPMS, beta-III tubulin and NeuN proteins in 661W and RGC-5 cells. Numbers below the blots indicate quantitation of expression in differentiated cells relative to undifferentiated cells adjusted with actin loading control.

661W cells were observed to be heterogeneous in size with prominent nuclei and some of these are elongated in shape. Upon adherence to the surface, 661W cells flatten out and start contacting the neighboring cells through their neuronal processes, usually attaching to multiple cells at a time. Treatment of 661W and RGC-5 cells with staurosporine for 24 hours resulted in differentiation into cells with prominent neuronal morphology^24^. Differentiated cells showed larger and more number of cytoplasmic projections/neurites, which could be visualized more clearly upon staining with acetylated tubulin-specific antibody (Fig. 4A,B). Brn3b, Brn3c and MAP2 proteins could be seen in the cell body as well as in the projections of differentiated cells (Fig. 4C and D). Expression of Thy1 and beta-III tubulin increased upon differentiation of 661W as well RGC-5 cells as seen by western blot (Fig. 4E). Increased expression of Thy1 and beta-III tubulin upon differentiation of RGC-5 cells has been reported previously also^25,27^. A marginal increase in RBPMS and NeuN was seen upon differentiation of 661W cells (Fig. 4E).

### Effect of expression of OPTN mutants on the survival of 661W cells

**Figure 5:**
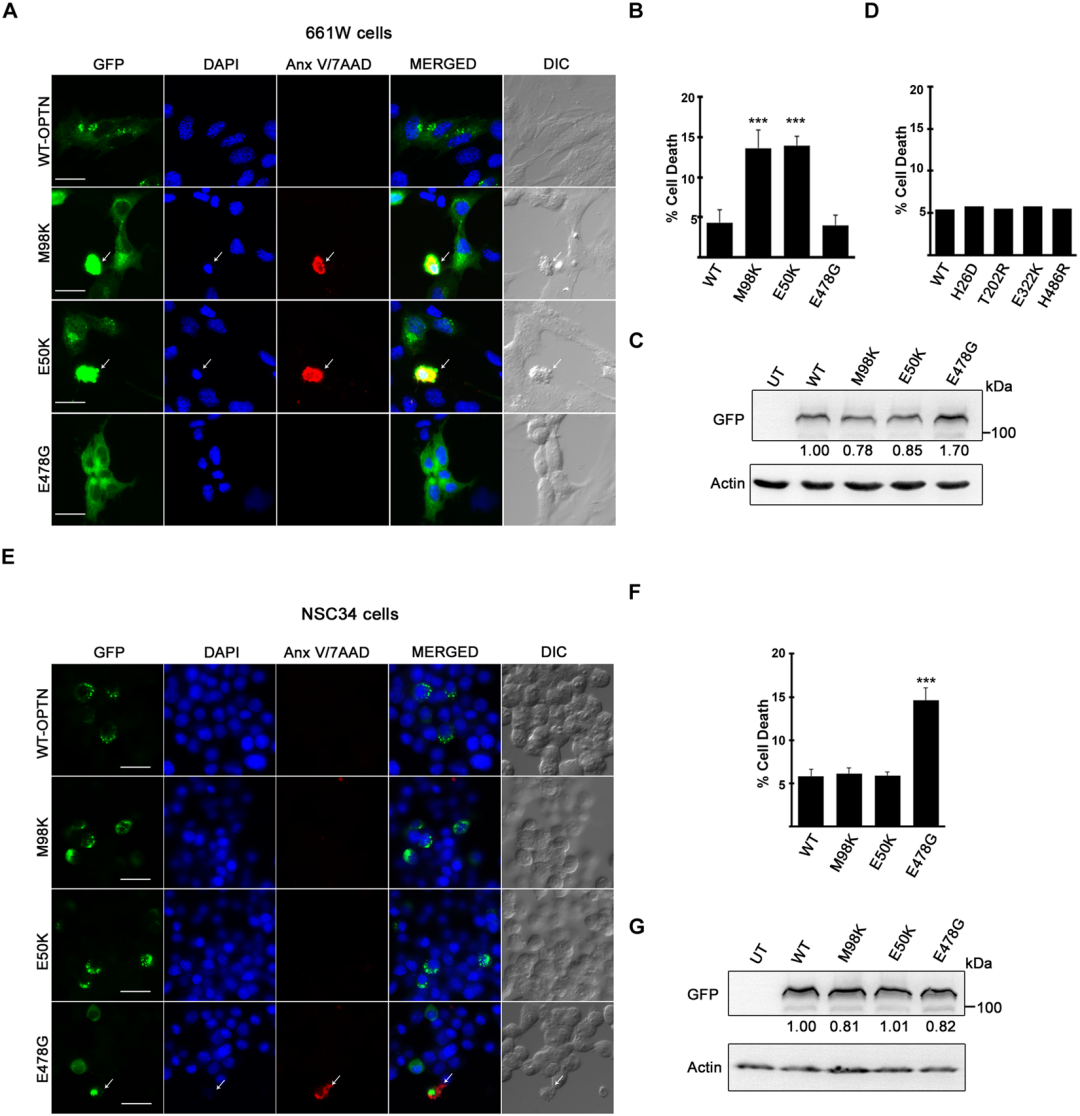
Effect of over-expression of OPTN and its mutants on the survival of 661W and NSC34 cells. (**A**,**E**) GFP tagged OPTN and its mutants were overexpressed by transfection in 661W and NSC34 cells grown on coverslips. After 32 hours of transfection, cells were stained for the detection of cell death. Panels show representative images of 661W (**A**) and NSC34 (**E**) cells expressing indicated proteins. Scale bar: 10 µm. (**B**,**F**) Quantitation of cell death induced by wild type OPTN and its mutants is shown in 661W and NSC34 cells, respectively. Data represent mean ± s.d. of percentage of GFP expressing cells showing Annexin V/7-AAD (Anx.V/7-AAD) staining after subtraction of cell death in non-expressing cells from three independent experiments done in duplicate. n = 6, ***p < 0.005. (**D**) Cell death induced by WT-OPTN and other glaucoma-associated mutants of OPTN quantitated using morphological criteria. Western blots show relative expression of OPTN and its mutants in 661W (**C**) and NSC34 cells (**G**). Actin was used as loading control.

In glaucoma, loss of vision occurs due to death of RGCs that leads to glaucomatous cupping of the optic nerve head^9,10^. The E50K mutant of OPTN is causatively associated with NTG in humans and it induces death of RGCs in transgenic mouse models^12,18,36–38^. NTG patients carrying OPTN mutations do not show any other pathology except glaucoma phenotype, although OPTN is expressed in many tissues^12,35^. This suggests that the damaging effects of glaucoma-associated mutants of OPTN are cell type specific. We examined the possibility of using 661W cell line as a cell culture model to study the damaging effects of OPTN mutants because these cells showed expression of several markers of RGCs. GFP tagged OPTN and its mutants were expressed by transfection of 661W cells and, after 32 hours, stained for AnnexinV/7AAD and DNA using an apoptosis detection kit (Fig. 5A). Compared to WT OPTN, two glaucoma-associated mutants of OPTN, E50K and M98K, induced significantly more cell death in these cells. However, an ALS-associated mutant, E478G did not induce more cell death than WT OPTN (Fig. 5B). Lack of induction of cell death by this mutant in 661W cells was not due to its lower expression as seen by western blot (Fig. 5C). Comparable results were obtained when cell death was determined using morphological criteria of apoptosis such as chromatin condensation, shrinkage of cells and membrane blebbing (data not shown). Expression of some other glaucoma-associated mutants of OPTN (H26D, H486R, T202R, E322K) did not induce more cell death than WT OPTN in 661W cells (Fig. 5D).

The effect of over expression of OPTN and its mutants was also examined in motor neuron-like cells, NSC34, a cell culture model for ALS^54^. In these cells, E50K and M98K mutants did not induce more cell death than WT OPTN, whereas E478G mutant induced significantly more cell death compared to WT OPTN, as determined by Annexin V/7AAD and DNA staining (Fig. 5E,F) or by using morphological criteria of apoptosis (data not shown). Induction of cell death by E478G mutant in NSC34 cells was not due to its higher expression than other mutants as seen by western blot (Fig. 5G). These results showed that glaucoma-associated mutants of OPTN, E50K and M98K, induced cell death selectively in 661W cells but not in NSC34 cells.

### Inhibition of M98K and E50K induced cell death in 661W cells

**Figure 6:**
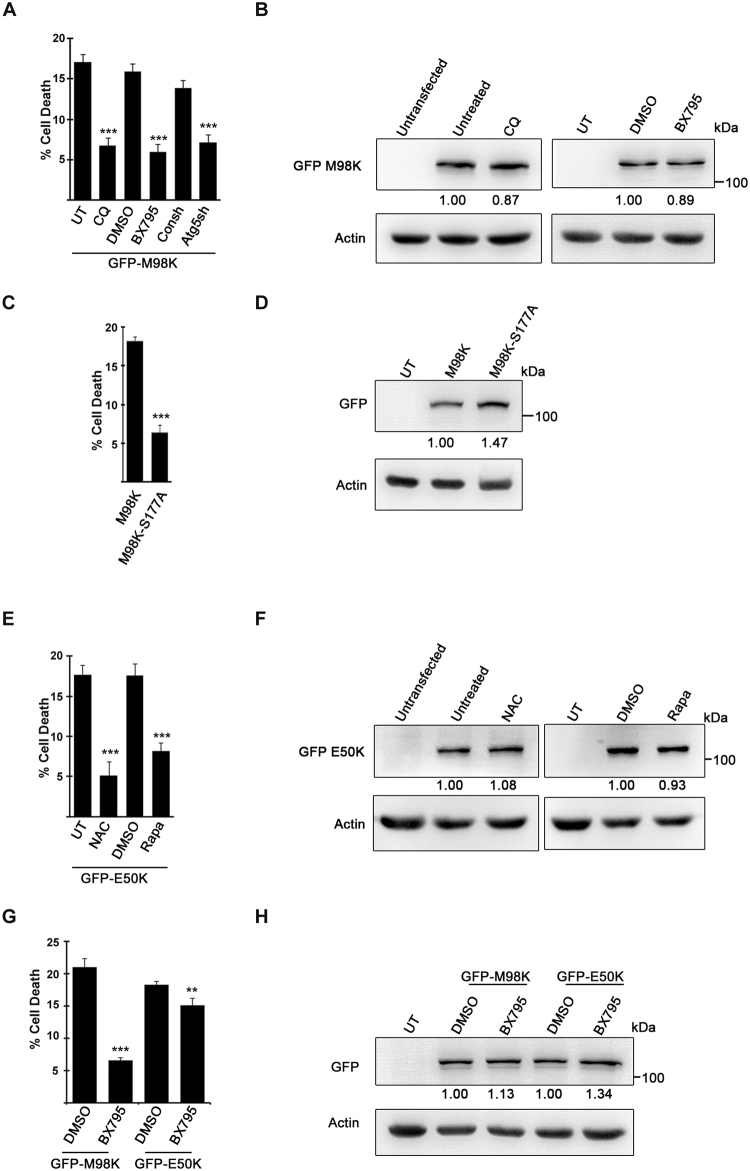
Mechanism of M98K-OPTN and E50K-OPTN induced cell death in 661W cells. (**A**) Effect of various inhibitors and *Atg5* knockdown on M98K-OPTN induced cell death. 661W cells transfected with GFP-M98K were treated with CQ (25 µM) or BX795 (1 µM) after 14 hours of transfection for 18 hours along with indicated controls and cell death was quantified based on Annexin V/7AAD positivity. 661W cells were cotransfected with GFP-M98K and control shRNA or *Atg5*shRNA and cell death was determined after 32 hours. (**C**) Effect of mutation of Ser177 to Ala in M98K-OPTN on induction of cell death in 661W cells. (**E**) Effect of antioxidant NAC and autophagy inducer rapamycin on E50K induced cell death. 661W cells were transfected with GFP-E50K. After 6 hours of transfection, cells were either treated with NAC (5 mM) for 26 hours or left untreated. GFP E50K transfected cells were treated with DMSO vehicle or rapamycin (1 µM) after 14 hours of transfection for 18 hours and cell death was quantified. (**G**) Effect of Tbk1 inhibitor on E50K-OPTN induced cell death. Treatment with BX795 (1 µM) was done as described for panel A. (**A**,**C**,**E**,**G**) Cell death data shown in bar diagrams represent mean ± s.d. of percentage of GFP expressing cells showing Annexin V/7-AAD (Anx. V/7-AAD) staining minus cell death in non-expressing cells from three independent experiments in duplicates. n = 6, ***p < 0.005 (**B**) Western blots showing expression of GFP-M98K upon CQ and BX795 treatment. Actin was used as loading control. (**D**) Western blot showing expression of the indicated mutants used for cell death experiment in C. (**F**) Western blots showing expression of GFP-E50K upon NAC and rapamycin treatment. (**H**) Western blots showing expression of GFP-M98K and GFP-E50K upon BX795 treatment. Actin was used as loading control. Numbers below the blots indicate quantitation of expression in treated cells relative to untreated cells adjusted with actin as loading control.

Mutations in *OPTN* and amplification of *TBK1* gene are associated with the same subtype of glaucoma (NTG)^12,14,18^. Phosphorylation of OPTN by TBK1 is involved in mediating autophagic function of OPTN. Previously we have shown that M98K-OPTN induced death of RGC-5 cells is mediated by autophagy and is dependent on Tbk1 kinase activity^29,39^. We examined the requirement of Tbk1 for M98K-OPTN induced death of 661W cells and found that this cell death was inhibited by Tbk1 inhibitor, BX-795 (Fig. 6A). Lysosomal inhibitor chloroquine (CQ) and knock down of Atg5 also inhibited M98K-OPTN induced death of 661W cells suggesting engagement of autophagy in cell death (Fig. 6A). Treatments with CQ and BX795 did not alter the expression levels GFP-M98K-OPTN as seen by western blotting (Fig. 6B). Mutation of Ser177 to Ala in M98K-OPTN reduced induction of cell death in 661W cells (Fig. 6C,D). E50K-OPTN induced death of 661W cells was inhibited by antioxidant, N-acetylcysteine (NAC) and autophagy inducer rapamycin (Fig. 6E) as was demonstrated earlier in RGC-5 cells^28,31^. Similar expression levels of GFP-E50K upon NAC and rapamycin treatment were confirmed by western blotting (Fig. 6F). These results suggest that the mechanisms of induction of cell death by M98K and E50K mutants in 661W cells are very similar to those reported previously in RGC-5 cells. Effect of Tbk1 inhibitor BX-795 was tested on E50K-OPTN induced death of 661W cells and it showed some inhibition at 1 µM concentration (Fig. 6G,H). Higher concentration (10 µM) of this inhibitor was toxic to these cells and therefore, could not be used. These results indicate that E50K-OPTN induced 661W cell death is partly dependent on kinase activity of Tbk1.

## Discussion

We have re-characterized 661W cells that have earlier been described as photoreceptor cell line but could be differentiated into neuronal cells. Our results suggest that 661W is a retinal ganglion precursor-like cell line, which expresses molecular markers of both RGCs and photoreceptor cells. This is not due to presence of mixed population of two types of cells, one expressing RGC-specific and the other expressing photoreceptor-specific markers, because all those markers that were tested by immunostaining (Brn3b, Brn3c, γ-synuclein, NeuN and Opn1mw), showed positivity in all the cells. In addition, these cells express nestin, a marker for neural precursor cells. During development of retina, multipotent RPCs give rise to RGCs and other types of retinal cells like photoreceptor cells. Gene regulatory networks controlled by transcription factors play a crucial role in determining the nature of the cells. Three transcription factors Atoh7 (Math5), Brn3b and Isl1 play a key role in the generation of RGCs from RPCs^55–57^. Atoh7 is an upstream regulator of RGCs, which is required for these cells to gain competence to produce RGCs^57^. Brn3b acts downstream of Atoh7 during RGC differentiation and it acts upstream of Brn3a^55,56^. Brn3b and Brn3c are also expressed in differentiated, mature RGCs. Expression of various markers indicates that 661W cells are RGC precursor-like cells.

In addition to using molecular marker analysis, we examined the effect of overexpression of two glaucoma-associated mutants, E50K and M98K, of OPTN in 661W cells. These mutants would be expected to induce death of RGCs selectively because they do not induce any other pathology in humans except NTG (which involves degeneration of RGCs and vision impairment)^12,35^. In fact, E50K mutant has been shown to induce RGC degeneration in transgenic mice^36–38^. We observed that the two glaucoma-associated OPTN mutants, E50K and M98K, but not an ALS-associated mutant, E478G, induced cell death in these cells but not in another neuronal cell line, NSC34, used as a model for ALS. Previously, E50K and M98K mutants have been shown to induce cell death selectively in RGC-5 cells but not in many other cell lines tested^31–33,39^. These observations suggest that 661W cells not only express several RGC-specific markers but also show RGC-like properties. Our results also showed that the effectors of cell death induced by OPTN mutants are similar in RGC-5 and 661W cells. Therefore, it is reasonable to suggest that these cells may be useful as a cell culture model for investigating the molecular mechanisms involved in induction of cell death relevant for glaucoma pathogenesis.

Glaucoma is characterized by cupping of the optic nerve head (known as glaucomatous cupping) that results due to degeneration of RGCs and their exons^9,10,18^. It has been reported that in addition to RGCs, cone photoreceptors are also lost during glaucoma in humans as well as in experimental animal models of glaucoma^6–8,36,58^. Therefore, it is not surprising that a cell line, 661W, which expresses markers of both RGCs and cone photoreceptors, shows selective induction of cell death by two glaucoma-associated mutants of OPTN.

RGC-5 cells were originally described as an RGC line obtained by immortalization of rat RGCs and was used by several groups^25^. It was later re-characterized and found to be of mouse origin^26,30^. In addition, expression of Thy1, Brn3 and neurofilaments was not seen in these cells, creating serious doubt about the identity of this cell line^27^. But these cells expressed several markers of neuronal cells such as Map1b, MAP2, PGP9.5 and beta III tubulin, and neural precursor marker nestin, suggesting, therefore that these cells are neuronal precursor cells. However, expression analysis for Brn3 family members was not carried out adequately. We carried out expression analysis of Brn3b and Brn3c proteins by western blotting using specific antibodies for these proteins and found that these transcription factors are expressed in RGC-5 cells. In addition, we observed that three other RGC-specific markers, RBPMS, Thy1 and γ-synuclein are expressed in RGC5 cells, although Brn3a is not expressed. Expression of γ-synuclein and Thy1 in RGC-5 cells has been reported previously also^25,42^. We also observed expression of NeuN, which is known to be expressed in RGCs but not in photoreceptor cells and used as RGC marker^36,50–52^. Expression of several markers of RGCs and neuronal cells, neuronal precursor marker nestin and cone specific opsin, suggests that RGC-5 cells, like 661W cells, are RGC precursor-like cells. This model of RGC-5 cells easily explains RGC-like property of selective induction of cell death in these cells by two glaucoma-associated mutants, E50K and M98K, of OPTN.

Although E50K and M98K mutants of OPTN when expressed in 661W (this study) and RGC-5 cells^31,39^ induce more cell death than wild type OPTN, some other glaucoma-associated mutants (H26D, H486R, T202R, E322K) did not induce this cell death in 661W (this study) as well as RGC-5 cells^31,39^. This indicates that E50K-OPTN and M98K-OPTN induce glaucoma possibly by directly inducing death of RGCs. Other glaucoma-associated mutants of OPTN (H486R, H26D,T202R, E322K) that do not induce death of 661W or RGC-5 cells may still be bona fide glaucoma-causing mutants, which possibly act by indirect mechanisms dependent on the niche *in vivo*, for example, by activating glial cells that may secrete cytotoxic molecules such as TNF alpha to induce RGC death. Another possibility is that these mutants may require cooperation from mutations in other genes to induce RGC death and glaucoma. Such a possibility is indicated by genetic studies, which indicate that glaucoma is a complex disease, which is affected by several interacting loci.

Overall, our results suggest that 661W cells are RGC precursor-like cells, which express several markers of RGCs and cone photoreceptor cells. These cells possibly represent a developmental stage just upstream of differentiated RGCs, as shown by expression of several molecular markers. This conclusion is supported by RGC-like property of selective induction of cell death by glaucoma-associated mutants of OPTN in these cells but not in other neuronal cells. Therefore, we suggest that these cells can be utilized for exploring the molecular mechanisms of RGC degeneration associated with glaucoma pathogenesis. We also show that RGC-5 cells express the same RGC-specific and other molecular markers as seen in 661W cells.

## Methods

### Cell culture, transfection and differentiation

An early passage (P12) of photoreceptor cell line 661W was provided by Dr. Muyyad R. Al Ubaidi^22^ in 2015 and all the experiments with this cell line were carried out using passage 16–30 cells. RGC-5 cells were provided by Dr. Neeraj Agarwal^25^. 661W, RGC-5 and NSC34 cells^54^ were grown in DMEM containing 10% fetal calf serum, penicillin and streptomycin in presence of 5% CO_2_ at 37 °C. Transient transfections were carried out using Lipofectamine 2000 in accordance with manufacturer’s protocol. Qiagen plasmid miniprep kit was used to purify the plasmids for transfection. For differentiation, cells were grown on coverslips for 24 hours and then treated with DMEM supplemented with 316 nM of Staurosporine and 10% fetal calf serum. Control cells were treated with solvent DMSO (0.1%). After 24 hours of treatment cells were fixed.

### Isolation of RNA and proteins from mouse retina

Approval of the Institutional Animal Ethics Committee of the Centre for Cellular and Molecular Biology, India, was taken for using mouse tissues. All animal experiments were performed in accordance with the relevant guidelines and regulations. Mice were euthanized and immediately eyeballs were removed and retina was dissected. Skull was cut open and whole brain tissue was collected. Retina and brain tissues were homogenized in liquid nitrogen and re-suspended in either Trizol for RNA isolation, or sample buffer for SDS-PAGE for protein analysis. Retina and brain were used as positive controls.

### cDNA synthesis and RT-PCR

RNA was isolated from cells grown in 35 mm dishes or tissue samples using TRIzol reagent (Invitrogen, Cat no 15596026) according to the manufacturer’s protocol. cDNA was made from 2 μg RNA using Superscript III cDNA synthesis kit (Invitrogen, Cat no 18080051). PCR was performed using Q5 master mix (Cat No M0494S), from NEB as per the manufacturer’s protocol. CD1 mouse retina was used as a positive control for this experiment. The sequence of primers used for PCR are as follows-OPN1MW (FP: GATTCTGGTGAACTTGGCAG, RP: ATGCGTGTCACCTCCTTCT), Nestin (FP: CGGGAGAGTCGCTTAGAGG, RP: CTTGGGGTCAGGAAAGCCAA) and GADPH (FP: ACCACAGTCCATGCCATCAC, RP: TCCACCACCCTGTTGCTGTA).

### Expression vectors and reagents

GFP tagged optineurin expression constructs WT-OPTN, E50K, M98K, M98K-S177A and E478G cloned in pEGFP-C3 (Clontech 6028–1), have been described previously^39^. Expression plasmids for WT-OPTN and its mutants H26D, H486R, T202R and E322K with HA tag have been described earlier^31,39^. TBK1 inhibitor- BX795 (Cat no. 204001) was purchased from Calbiochem. Chloroquine (Cat. No.6628), Rapamycin (Cat. No. R0395) and Staurosporine (Cat. No. S5931) were procured from Sigma.

### Indirect immunofluorescence and western blotting

661W and other cells were grown on coverslips and after the required treatment or transfection, fixed with 3.7% formaldehyde for 10 min at room temperature. For staining with γ-synuclein antibody the cells were fixed with methanol at −20 °C for 6 minutes. Fixed cells were then washed, permeabilized, stained with appropriate primary antibodies and incubated overnight at 4 °C. The dilutions of various antibodies used are given in Table 1. After washing, respective secondary antibodies were added and the cells were incubated at 37 °C for 1 hour in the dark. Coverslips were mounted in PBS containing 90% glycerol, 1 mg/ml para-phenylenediamine and 0.5 mg/ml 4′,6-diamino-2-phenylindole (DAPI), and observed under Axioimager Z.1 (Zeiss) fluorescent microscope × 40/0.75NA objective. Images were captured using AxioVision software.

**Table 1:** Antibodies and their dilutions used in Western Blotting (WB) and Immunofluorescence (IF).

Sr. No.	Antibody	Company	Catalogue Number	Host	Dilution for WB	Dilution for IF
1	RBPMS	ProSci	29–239	Rabbit	1:1000	—
2	Thy1	R & D	mab7335	Rat	1:1000	—
3	Thy1	CST	13801 S	Rabbit	1:1000	—
4	Thy1	Santa Cruz	sc-53116	Mouse	1:1000	—
5	γ Synuclein	Abcam	Ab55424	Rabbit	—	1:150
6	Brn3b	ProteinTech	55042-1-AP	Rabbit	1:1500	1:50
7	Brn3c	ProteinTech	21509-1-AP	Rabbit	1:1800	1:100
8	Nestin	Santa Cruz	sc-21248	Goat	1:1500	1:200
9	OPN1MW	Santa Cruz	sc-30022	Rabbit	1:1200	1:100
10	NeuN	Abcam	ab104224	Mouse	1:3500	1:1000
11	β-III Tubulin	Abcam	Ab81207	Rabbit	1:1000	1:100
12	MAP2	CST	4245 S	Rabbit	1:1000	1:100
13	Ac-Tubulin	Sigma	T7451	Mouse	—	1:500
14	GFAP	Invitrogen	180021	Mouse	1:1000	—
15	GFP	Santa Cruz	Sc9996	Mouse	1:1000	—
16	Actin	Millipore	1501	Mouse	1:10000	—
17	Anti Rabbit HRP	Amersham	NA934	Donkey	1:5000	—
18	Anti Mouse HRP	Amersham	NA931	Sheep	1:5000	—
19	Anti goat HRP	Santa Cruz	sc-2020	Donkey	1:5000	—
20	Anti rat HRP	Pierce	31470	Goat	1:5000	—
21	Mouse Alexa 488	Invitrogen	832723	Goat	—	1:1200
22	Rabbit Cy3	Millipore	AP132C	Goat	—	1:1500
23	Goat Cy3	Jackson IR	305-035-003	Rabbit	—	1:500

Western blotting was carried out as described earlier^59^. Protein lysates were resolved by SDS-PAGE and transferred on PVDF membrane. The membrane was then blocked by 5% Blotto in TBST (10 mM Tris-Cl, pH 7.6, 150 mM NaCl, 0.1% Tween 20), followed by overnight incubation in primary antibody. After washing, the membrane was incubated in horseradish peroxidase (HRP) conjugated secondary antibodies for 1 hour and the blot was then developed by using ECL reagents.

### Cell Death Assay

Cell death assays were carried out as described earlier^60^. Cells were seeded on coverslips and transiently transfected with GFP tagged OPTN and its mutants. After 32 hours of transfection, apoptosis assay was performed using GFP certified apoptosis assay kit (Enzo Life Sciences, Cat no. ENZ-51002-100) as per the manufacturer’s protocol. Cells showing either or both Annexin V and 7-aminoactinomycin D (7-AAD) staining were scored positive. At least 200 GFP expressing and 1000 GFP non-expressing cells were scored from each coverslip. The data shown represent mean ± sd from three experiments performed in duplicates. For cell death based on morphological apoptotic criteria, features like membrane blebbing, cytoplasmic shrinkage, nuclear condensation and loss of refractility were used to score dead cells after fixation in 3.7% formaldehyde^31^.

### Statistical Analysis

Bar diagrams represent mean ± sd values. To determine statistically significant difference in means two tailed student’s t-test was used.

### Data availability statement

All data generated or analysed during this study are included in this published article.
